# Scotland's Core Trainees & Specialty Doctors: A Collective Report on Opinions and Attitudes Towards the Current Limits on Higher Training in Psychiatry

**DOI:** 10.1192/bjo.2024.284

**Published:** 2024-08-01

**Authors:** Ailsa Bruce, Ewan Mahony, Arwa Elawad, Siobhan Connelly, Rachel Ball

**Affiliations:** 1 NHS Lothian, Edinburgh, United Kingdom; 2 NHS Tayside, Dundee, United Kingdom; 3 NHS Greater Glasgow and Clyde, Glasgow, United Kingdom; 4 NHS Grampian, Aberdeen, United Kingdom

## Abstract

**Aims:**

1. To provide Scotland-wide data on Core Trainees’ motivations, their future plans and the barriers to applying for Higher Training.
2. To raise awareness of any collective issues.
3. To provide recommendations to the Royal College of Psychiatrists, NHS Education for Scotland (NES) and the Scottish Government based on the results.

**Methods:**

A Microsoft Forms survey was emailed to all 176 Core Trainees in Scotland through regional PTC representatives in East, West, South East and through the Core Training Programme Director in the North. Speciality doctors who were post Core Training, and waiting to apply for Higher Training, were identified by snowball sampling and were also emailed a link to the survey. Data was collected between 26/10/23 and 21/11/23.

**Results:**

- All regions in Scotland and all levels of training were well represented by trainee response rates. Trainee participation was high with 90 doctors responding from across all areas in Scotland and all levels of training.
- 83.3% of trainees feel that the current availability of Higher Training posts is affecting morale and motivation in psychiatry.
- 96% of trainees plan to enter Higher Psychiatry Training and the majority of trainees (63%) want to enter Higher Training directly from Core Training. The availability of their chosen Higher Training post was the number one reason for not wanting to enter Higher Training directly.
- Less than full time working is increasing and likely to increase further (nearly 29% of participants are currently LTFT. 30% definitely plan to do some of their Higher Training LTFT and a further 34% are considering it).
- The majority of trainees (70%) wish to continue training in their current region. Trainees may be lost from Scotland if they are unable to secure a training post in their chosen region (27% of those considering another region would consider leaving Scotland). Those who would consider leaving Scotland came from all regions – of the 27%: 22% were East, 26% North, 26% South East and 26% West. Second choice regions for consideration remain those that have the most filled posts in Scotland (27% would consider South East Scotland, 22% West, 15% East and 9% North).
- Participants included lengthy and detailed responses to a free text box at the end of the survey titled “Do you have any additional comments” with several recurring themes. These included less than full time not being accounted for in the overall Higher Training numbers, difficulties in moving region, feeling stressed and demoralised by the application process, feeling undervalued and considerations around leaving Scotland.

**Conclusion:**

1. The primary obstacle preventing core trainees from progressing to Higher Training, as identified by them consistently across regions, is the scarcity of available Higher Training posts across regions, relative to the number of Core Trainees finishing their Core Training.
2. The ongoing increase in less than full time working, with two-thirds of trainees considering pursuing some of their Higher Training on a less than full time basis, will further delay the release of training numbers and therefore growth of consultant numbers without full time equivalent numbers.
3. Trainees may be lost from Scotland. The majority of trainees settle in their Core Training region and there are several reasons that moving may be difficult. Of those who would contemplate relocation, 27% would consider leaving Scotland and the main regions in Scotland that would be considered as alternatives already have the highest fill rates.

